# Psychosocial Impact of Celiac Disease on Primary Caregivers of Children in Jordan: A Cross-Sectional Study

**DOI:** 10.3390/children13040533

**Published:** 2026-04-12

**Authors:** Ala’a Al-Dala’ien, Nedal Alnawaiseh, Khitam Al-Refu, Assal H. Al-Btoush

**Affiliations:** 1 Department of Pediatrics, Faculty of Medicine, Mutah University, Al-Karak 61710, Jordan; 2 Community Medicine and Public Health Department, Faculty of Medicine, Mutah University, Al-Karak 61710, Jordan; 3 Department of Internal and Forensic Medicine, Faculty of Medicine, Mutah University, Al-Karak 61710, Jordan; alrefukhi@mutah.edu.jo; 4 Faculty of Medicine, Mutah University, Al-Karak 61710, Jordan; 120221503265@mutah.edu.jo

**Keywords:** celiac disease, primary caregivers, psychosocial burden, quality of life, gluten-free diet, Jordan

## Abstract

**Highlights:**

**What are the main findings?**

- Caregivers of children with celiac disease in Jordan experience moderate to moderately high psychosocial burden.
- Younger age, lower income, and lower education were associated with higher caregiver burden.

**What is the implication of the main finding?**

- Family-centered support and targeted education programs are needed to improve caregiver well-being.
- Enhancing the availability and accessibility of gluten-free food can reduce both the psychological and financial burden on caregivers.

**Abstract:**

**Background:** Celiac disease (CD) is a chronic immune-mediated disorder requiring lifelong adherence to a strict gluten-free diet (GFD). Similar to chronic pediatric disorders, primary caregivers are susceptible to significant psychosocial burden, as CD management relies heavily on them. However, evidence regarding their psychological well-being in Jordan remains limited. **Objective:** This study aimed to investigate the psychosocial burden on Jordanian caregivers of pediatric CD patients and examine associations with clinical and sociodemographic factors. **Methods:** This cross-sectional study recruited Jordanian caregivers of pediatric CD patients with biopsy-confirmed diagnosis from September to December 2025, and all patients had followed a GFD for at least six months. The Arabic version of the Celiac Disease Parent/Caregiver Quality of Life Questionnaire (CDPCA-QoL) was distributed online, alongside questions on child and caregiver sociodemographic factors. Descriptive and inferential analyses, including *t*-tests, ANOVA, correlation, and multiple linear regression, were performed. **Results:** A total of 198 caregivers participated (mean age 39.5 ± 9.8 years; 85.9% female). Overall burden ranged from moderate to moderately high. Parental worries had the highest mean score (3.95 ± 0.66), followed by emotional (3.48 ± 0.73) and social functioning (2.96 ± 0.99). Younger age, lower education, and lower income were significantly associated with greater burden (*p* < 0.05). Caring for more than one affected child increased emotional and social strain, whereas longer disease duration was linked to lower parental worry. No associations were found with caregiver gender, marital status, employment, or child characteristics. **Conclusions:** Jordanian caregivers experience considerable psychosocial burden, emphasizing the need for targeted psychosocial and family-centered support.

## 1. Introduction

Celiac disease (CD) is an immune-mediated systemic disorder triggered by ingestion of gluten in genetically susceptible individuals and is managed primarily by lifelong adherence to a strict gluten-free diet (GFD) [1,2]. It is increasingly recognized in children and adolescents, with growing diagnosis rates in the Middle East and North Africa, including Jordan [3]. Although advances in diagnosis and management have improved clinical outcomes, CD remains a chronic condition requiring continuous dietary monitoring and vigilance against unintentional gluten exposure, placing substantial responsibility on families [4].

In pediatric populations, CD presents with both gastrointestinal and extra-intestinal symptoms and can negatively affect growth, academic performance, and social relationships. These challenges contribute to reduced health-related quality of life (HRQoL) in affected children, which may extend to their caregivers [5]. Caregivers commonly report elevated anxiety around dietary adherence and cross-contact, reduced social participation (e.g., reluctance to attend social events or school functions), increased time and financial costs associated with specialty foods, and a sense of constant vigilance that can impair well-being [4,6,7,8].

The concept of caregiver burden can be understood within the stress–coping framework, which posits that psychological outcomes result from the interaction between stressors (e.g., disease management demands), individual appraisal, and available coping resources. In the context of pediatric CD, ongoing dietary restrictions, fear of accidental gluten exposure, and social limitations represent chronic stressors that may exceed caregivers’ coping capacity, leading to psychological distress and reduced quality of life. This framework provides a useful lens for examining how disease-related demands translate into measurable psychosocial burden.

Systematic and narrative reviews indicate that caregiver well-being in pediatric CD has been insufficiently studied, but existing work points to increased caregiver stress, anxiety and reduced quality of life compared with parents of healthy children [4]. Empirical studies from different cultural contexts (including quantitative burden measures and qualitative explorations of lived experience) highlight common themes—fear about accidental gluten ingestion, disruption of family mealtimes, stigma and social isolation—while also showing that local food cultures, healthcare structures and social supports shape the intensity and expression of burden [8,9,10].

Available evidence from Jordan documents the reduction of HRQoL as well as a reduction in growth and academic performance in the pediatric population affected by CD, emphasizing its social and clinical effects on the Jordanian population [5]. When compared globally, marked differences are observed between high- and low-income countries in terms of CD services such as gluten-free products, dietitian support and diagnosis accessibility, which in turn increase caregivers’ responsibilities in countries such as Jordan [11].

To our knowledge, no study has specifically investigated Jordanian caregivers of pediatric CD patients in terms of psychosocial stress. The majority of previous studies were patient centered, exploring aspects such as diagnosis, HRQoL, and disease prevalence. However, literature focused on primary caregivers is still limited [5,12].

Understanding caregiver burden has both scientific and practical importance. Primary caregivers—typically parents—are central to successful disease management in children: they ensure dietary adherence, negotiate social and educational accommodations, and provide day-to-day emotional support. Caregiver distress can therefore adversely affect child health outcomes, adherence to a GFD and family functioning. Moreover, identifying the specific psychosocial domains that are most affected in Jordanian caregivers (anxiety, depression, social functioning, financial strain, stigma, or family dynamics) is a prerequisite for culturally appropriate interventions, educational strategies and health-service planning [4,8,10].

This study aims to fill the gap by assessing the psychosocial impact of pediatric CD on primary caregivers in Jordan. Specifically, this study measures the psychosocial burden on primary caregivers and also investigates its relationship with sociodemographic factors and the child’s CD variables. This study is relevant to the present literature since it generates a local database that can facilitate in future evidence-driven recommendations that can improve caregivers’ well-being and lead to a family-centered care model for Jordanian pediatric CD patients.

Accordingly, we hypothesized that caregivers of children with celiac disease in Jordan would experience a measurable psychosocial burden and that this burden would be associated with key sociodemographic and clinical factors such as caregiver age, education level, income, and disease-related characteristics.

## 2. Material and Methods

### 2.1. Study Design and Subjects

This research adopts a cross-sectional descriptive design to evaluate the psychosocial impact of caring for children with celiac disease on their primary caregivers in Jordan. This design is appropriate for assessing psychological, emotional, and social outcomes at a single point in time and for exploring associations between caregiving burden and relevant demographic or contextual factors. In this study, we distributed a web-based version of our questionnaire to the caregivers via Instagram, WhatsApp, and Facebook platforms. Reaching the primary caregivers of children was possible via the Jordanian Celiac Disease Care Providers Society. All twelve Jordanian governorates were included.

Eligibility criteria were clearly defined to ensure inclusion of primary caregivers who (1) had <18-year-old child with biopsy-confirmed diagnosis of celiac disease who has been on a gluten-free diet for at least 6 months residing in Jordan; (2) provided informed consent to participate in this study; and (3) could read, write, and speak in Arabic to participate in this study. Caregivers were recruited from different geographic regions in Jordan (urban and rural) to ensure diversity and generalizability.

All participants received information about the study objectives and procedures and provided informed consent prior to participation. This study was conducted from September to December 2025.

### 2.2. Sample Size and Justification

The sample size was determined based on methodological recommendations for cross-sectional studies employing multivariable analysis. Because the study aimed to examine the association between psychosocial burden and multiple caregiver- and child-related factors, a total of 16 independent variables were initially considered as potential predictors. A commonly accepted recommendation is to include at least 10–15 participants per predictor variable to obtain stable and reliable estimates, suggesting a required sample size of 160–240 participants. In addition, based on the criteria proposed by Tabachnick and Fidell for multiple regression analysis (N ≥ 50 + 8 m), a minimum sample of 178 participants was required. The achieved sample of 198 caregivers meets these criteria, indicating that the sample size was adequate to support the planned descriptive and inferential analyses, including *t*-tests, ANOVA, correlation, and multiple linear regression. Furthermore, the final regression models retained fewer predictors, further supporting the stability and reliability of the estimates. This sample size is also comparable to those reported in similar studies evaluating caregiver burden in pediatric chronic conditions.

### 2.3. Data Collection Tool

Through a self-reported questionnaire, we collected the participants’ sociodemographic and clinical data including caregiver’s age, sex, marital status, education level, relationship to the child, self-diagnosis of celiac disease, number of family members, family history of celiac disease, having other children with celiac disease, employment status, average monthly income, along with their children’s age, sex, age at time of celiac diagnosis, time since diagnosis and history of other chronic diseases in addition to celiac disease.

The Celiac Disease Parent/Caregiver Questionnaire (CDPCA-QoL) by Pavia et al. [13] was used to collect data. This is a validated instrument designed specifically to assess the emotional, psychological, and social impact of managing celiac disease from the caregiver’s perspective.

Components of the CDPCA-QoL:

1. Emotional Functioning (Questions 1–10): Measures caregiver stress, anxiety, and emotional burden.
2. Parental Worry (Questions 11–20): Studies caregivers’ concerns regarding the child’s compliance with a GFD and subsequent long-term health and development.
3. Social Functioning (Questions 21–30): Assesses impact on relationships, social life, and stigma.

A 5-point Likert scale was used to record the answers from caregivers. The responses varied between Always (5), Almost always (4), Sometimes (3), Almost never (2), Never (1). Each final score had a possible range from 1–5 with higher scores suggesting poor QoL. The range of the scale was 4 points (5 − 1 = 4), the range was divided into three equal dividends, and the resultant three levels were classified according to the mean value of total scores for each question and item as follows:

1. Low psychosocial impact = 1 to 2.33.
2. Moderate psychosocial impact = 2.34 to 3.66.
3. High psychosocial impact = 3.67 to 5.

For this study, the CDPCA-QoL questionnaire was translated into Arabic for academic research purposes. The translation process followed standard forward–backward translation procedures and was reviewed by an expert panel to ensure linguistic and conceptual equivalence. The translated version was further checked for its accuracy and ease of conducting through the work of a pilot study including 16 primary caregivers of children with celiac disease, in order to evaluate the questionnaire questions, the difficulties that may face answering questions, knowing the time required to answer the questions of the questionnaire and to ensure that the questionnaire questions are appropriate for the participants. The data was checked and analyzed to identify any questions that are not clear or difficult to answer. The expert panel group also evaluated verification of content, translation process of the questionnaire, assessment of reliability, face validity and content validity.

To assess the construct validity of the Arabic version of the CDPCA-QoL questionnaire, an exploratory factor analysis (EFA) was conducted using principal component analysis with varimax rotation. The suitability of the data for factor analysis was evaluated using the Kaiser–Meyer–Olkin (KMO) measure of sampling adequacy and Bartlett’s test of sphericity. The KMO value was 0.89, indicating excellent sampling adequacy, while Bartlett’s test was statistically significant (χ^2^ = 3125.4, *p* < 0.001), confirming that the data were appropriate for factor analysis.

Factors were retained based on eigenvalues greater than 1 and inspection of the scree plot. A three-factor solution was identified, corresponding to the original domains of the questionnaire: emotional functioning, parental worry, and social functioning.

Factor loadings ≥0.40 were considered significant. The majority of items loaded strongly on their respective domains, with minimal cross-loading observed. These findings support the construct validity of the Arabic version of the CDPCA-QoL questionnaire.

In addition, the Arabic version of the CDPCA-QoL questionnaire demonstrated excellent internal consistency. Cronbach’s alpha values were 0.807 for the emotional functioning domain, 0.800 for the parental worry domain, and 0.920 for the social functioning domain. The overall scale showed a Cronbach’s alpha of 0.927, indicating high reliability of the Arabic version of the questionnaire in this population.

### 2.4. Data Analysis

All data analyses were conducted using the Statistical Package for the Social Sciences (version 28). Descriptive statistics (means, standard deviations, frequencies, and percentages) were calculated to describe caregiver characteristics and study variables. Correlation analyses were used to assess relationships between continuous variables. Independent samples *t*-tests and one-way ANOVA were conducted to compare mean differences across groups. Multiple linear regression analysis was performed to identify significant predictors of the impact of celiac disease on caregivers. A *p*-value of ≤0.05 was considered to indicate statistical significance.

### 2.5. Ethical Approval and Informed Consent

This study was granted approval by the Institutional Review Board (IRB) at Mutah University (reference number: 61025, dated on 24 August 2025). All participants were asked to provide written informed consent after they were informed that their participation was voluntary, anonymous and that they had the right to withdraw from the study at any time without consequence.

## 3. Results

A total of 198 primary caregivers of children with celiac disease were included in the analysis, with no missing data. The majority were female (85.9%) and mothers of the affected child (82.8%), with a mean age of 39.5 ± 9.8 years. Among children, 63.6% were female, with a mean age of 11.1 ± 4.7 years and a mean age at diagnosis of 8.5 ± 5.2 years. More than half of the children (53.0%) had been diagnosed for more than three years, and 44.4% had at least one additional chronic condition (Table 1).

Caregiver psychosocial burden, assessed using the CDPCA-QoL, ranged from moderate to moderately high. The highest mean score was observed in the parental worries domain (3.95 ± 0.66), followed by emotional functioning (3.48 ± 0.73) and social functioning (2.96 ± 0.99).

None of the three domains had statistically significant differences when compared to the caregiver’s gender, marital and employment status, caregiver diagnosis of celiac disease, family history of CD, child’s gender, age or existence of comorbid conditions (*p* > 0.05). On the contrary, being a caregiver responsible for more than one CD child was significantly associated with higher scores in both the emotional and social functioning domains (*p* = 0.003, *p* = 0.002 respectively) (Table 2).

As shown by Spearman’s correlation, caregiver’s age was inversely related to all three domains (*p* < 0.001), which indicates higher levels of psychosocial burden amongst younger caregivers. Monthly income status was inversely correlated with both emotional functioning and parental worry domains (*p* < 0.001). No significant correlations were found between burden scores and family size, child age, or age at diagnosis (Table 3).

One-way ANOVA demonstrated significant differences in emotional burden according to educational level (*p* < 0.001), with caregivers of lower educational attainment reporting higher scores. Parental worries differed significantly by relationship to the child (*p* = 0.035) and time since diagnosis (*p* = 0.008), with lower worry scores among caregivers of children diagnosed for more than three years. Caregiver age groups differed significantly across all domains, with younger caregivers reporting higher psychosocial burden (Table 4).

In multiple linear regression analyses, younger caregiver age consistently predicted higher emotional, worries, social, and total psychosocial impact scores (all *p* < 0.01). Lower income and lower educational level predicted higher emotional and worries scores, while having more than one affected child predicted higher social and total psychosocial impact. Female gender was a significant predictor of higher emotional burden (Table 5).

In addition to statistical significance, the magnitude of the observed associations was considered. The regression coefficients (B values) indicate that caregiver age showed a consistent and meaningful negative association with psychosocial burden across all domains, suggesting a practically relevant effect. Similarly, education level and income demonstrated modest but meaningful effects on emotional and worry domains, while having more than one affected child showed a noticeable impact on social burden.

## 4. Discussion

To our knowledge, this is the first study conducted in Jordan to explore the psychosocial burden experienced by primary caregivers of children with celiac disease (CD). Overall, caregivers reported a moderate to moderately high psychosocial burden, with parental worries emerging as the most affected domain, followed by emotional functioning and social functioning. Such results highlight the multidimensional nature of pediatric CD, where the burden extends beyond the child to affect the caregiver’s psychological, emotional, and social well-being. This may reflect the central role caregivers play in managing strict dietary adherence in environments where institutional support is limited, placing primary responsibility on the family.

In this study, the worries domain achieved the highest mean score compared to the other two domains, indicating that it is a main source of parental stress. The parental worries domain in CDPCA-QoL addressed concerns related to the patient’s compliance with a GFD, sufficient nutritional intake, long-term complications, and accidental gluten intake. This is consistent with the available literature, in which the major caregiver stressors were their concerns of long-term complications and dietary vigilance [4,14]. However, in the Jordanian context, these challenges may be amplified by limited regulatory enforcement of food labeling, reliance on imported gluten-free products, and variability in school-level awareness. Collectively, this creates an environment where caregivers must remain constantly vigilant, thereby intensifying anxiety and perceived burden.

These factors have already been identified in other middle-income countries [15,16,17,18]. Another well-known major contributor to caregivers’ psychosocial burden is financial instability and economic stress [8,19,20].

Moderate to moderately high burden scores were also observed in the emotional domain, reflecting substantial psychological strain among caregivers. This may be attributed not only to the chronic nature of CD and the lifelong commitment to dietary management but also to the lack of structured psychosocial support services for caregivers in Jordan. Healthcare systems in similar settings often prioritize the clinical management of the child, with less emphasis on caregiver mental health. Consequently, caregivers may experience sustained stress, anxiety, and emotional exhaustion, consistent with studies showing higher rates of anxiety and depression among parents of children with CD compared to controls [10,21]. This supports the broader understanding that chronic diseases requiring continuous lifestyle modification impose persistent emotional demands on caregivers, regardless of disease severity [22,23].

Caregivers in this study reported a moderate impact on social functioning, which is consistent with prior studies showing that CD imposes social restrictions on family life, including limitations on eating outside the home, attending social gatherings, and traveling [24,25,26]. In Jordanian society, which is largely collectivist, food plays a central role in social interaction, hospitality, and family cohesion. Shared meals are frequent, and declining food may carry social implications. This cultural context can complicate adherence to a strict GFD, requiring caregivers to navigate social expectations while ensuring dietary safety. Interestingly, social burden was the least affected domain. This may suggest the development of adaptive coping strategies, such as modifying participation in social events or relying on close family networks who may be more understanding and accommodating. It may also reflect normalization of these challenges over time within tightly knit family structures.

Some studies proposed that female caregivers experience higher burden levels than males do [8,26]. In our study, however, we could not identify a significant difference between in the three domains between genders, similar to what Pavia et al. have reported [13]. Such results might indicate equal caregiving burden between both genders. Another reason may be the increase in responsibility sharing amongst Jordanian parents. Alternatively, it may reflect sociocultural influences on reporting, where male caregivers may underreport emotional distress due to societal expectations regarding emotional expression. Notably, caregiver gender emerged as a significant predictor in the regression model, suggesting that its influence may be more complex and context-dependent when accounting for other variables.

Interestingly, we identified a clear pattern of an inverse relationship between caregivers’ age and burden across all three domains. Hence, being a younger caregiver is associated with significantly higher levels of social burden, parental worries and emotional distress. Our results are consistent with the available data on chronic pediatric disorders. This burden difference may be attributed to older caregivers’ skills learned throughout their career such as critical thinking, emotional intelligence and coping mechanisms [27,28]. In the Jordanian context, younger caregivers may also face additional pressures, including early parenthood, financial instability, and limited experience navigating healthcare systems. Furthermore, cultural expectations and responsibilities within extended family structures may place additional strain on younger parents, intensifying the overall caregiving burden.

Parental worries were significantly lower among caregivers of children diagnosed for more than three years, suggesting gradual adaptation and increased confidence in managing the GFD over time, which was similarly noticed in a study from Turkey [8]. However, the lack of meaningful variation in emotional and social well-being based on how long the diagnosis had been established indicates that some elements of caregiver strain remain unchanged over time. Even with greater experience and familiarity with the condition, these challenges may continue, highlighting the long-term and ongoing nature of stress associated with celiac disease [29].

A higher emotional burden was associated with a lower educational level, emphasizing that health literacy is essential in the management of CD. Highly educated caregivers are more capable of reading and understanding food labels, searching for nutrition information, and having effective communication with healthcare providers. All these factors play a central role in effective CD management [27].

A negative correlation has been found between parental emotional burden and family income, which indicates that maintaining a GFD might carry economic challenges. This also aligns with a study from Brazil [13]. High caregiver stress related to financial limitations due to challenges related to the cost and accessibility of gluten-free food is notable in Jordan. These findings are consistent with previous studies, which highlighted a substantial financial burden of gluten-free diets, especially in regions where this kind of food is not easily accessible or not subsidized [18,19]. Although the association between income and social functioning was not significant, it is noticeable that financial resources enable social engagement and flexible dietary choices.

The number of children affected by CD is a primary player in this aspect; the more children, the greater the psychosocial burden. This has huge stressful consequences on the caregivers, who have to maintain a strict gluten-free diet and are constantly alert to dietary contamination and also worried about long-term consequences for their children’s health. This is consistent with caregiving research that showed burden increases with caregiving intensity and number of care recipients [30].

Surprisingly, our study has shown that age, gender and comorbid conditions of children are not associated with caregiver burden, in contrast to other studies that found that younger children or those with comorbid conditions resulted in more distress in caregivers [13]. The primary caregiver distress in CD was due to the difficulty of constant attention to their children’s diet and lifestyle, regardless of age.

This discrepancy may be explained by cultural differences, variations in healthcare systems, and differences in study populations. In the Jordanian context, caregiver burden may be more strongly influenced by external factors such as financial constraints, limited availability of gluten-free products, and challenges related to dietary management, rather than child-specific clinical characteristics.

### 4.1. Implications for Practice and Policy

Our study highlights the importance of implementing a family-centered approach to manage pediatric CD in Jordan. Targeted support should be directed to younger caregivers, those with lower educational levels and low income. Herein, we suggest developing structured education programs and establishing support groups as well as improving the accessibility of gluten-free food which will reflect positively on the caregiver psychological and financial burden. Furthermore, we suggest conducting awareness programs in schools and communities which will reduce psychological stress in these families.

### 4.2. Strengths and Limitations

This study is the first to investigate in Jordan the psychosocial burden of primary caregivers of pediatric CD patients using CDPCA-QoL, which is a tool made specifically to measure CD’s burden on caregivers. Additionally, the study included a large sample size and was able to assess several psychosocial domains. However, since it is a cross-sectional study, it is hard to establish causality. Additionally, the use of a self-reported online questionnaire distributed through social media may introduce selection bias and limit the generalizability of the findings, as individuals who are more engaged, have higher awareness, or have better access to digital platforms may be overrepresented in the sample. We suggest following up caregivers over time via a longitudinal study to get a deeper insight into the cultural strategies to deal with CD.

## 5. Conclusions

In conclusion, caregivers of children with celiac disease in Jordan experience a considerable psychosocial burden, largely driven by concerns related to dietary management. This burden is significantly associated with younger caregiver age, lower educational level, limited income, and having more than one affected child, identifying vulnerable groups that may benefit from targeted support.

These findings underscore the importance of integrating caregiver-focused strategies into routine pediatric celiac disease care. Clinically, structured, dietitian-led educational interventions and regular psychosocial assessment should be incorporated into standard management to improve caregivers’ coping capacity and dietary adherence. A multidisciplinary approach involving healthcare providers and mental health professionals may further reduce caregiver distress.

At the policy level, improving the availability and affordability of gluten-free products remains essential. In addition, implementing school-based support systems and increasing awareness among educators may help mitigate caregiver anxiety related to disease management outside the home.

Future research should evaluate culturally appropriate interventions and longitudinal outcomes to better understand and address caregiver burden in similar settings.

## Figures and Tables

**Table 1:** Sociodemographic and Clinical Characteristics of Caregivers and Children with Celiac Disease (N = 198).

Caregivers Characteristics	N (%)
**Gender**	
Male	28 (14.1)
Female	170 (58.9)
**Marital status**	
Married	186 (93.9)
Not married	12 (6.1)
**Education level**	
School	84 (42.4)
Bachelor degree	62 (31.3)
Diploma	36 (18.2)
Higher studies	16 (8.1)
**Employment status**	
Employed	63 (31.8)
Not employed	135 (68.2)
**Relationship to the child**	
Father	20 (10.1)
Mother	164 (82.8)
Others	14 (7.1)
**Caregivers with celiac disease diagnosis**	
Yes	34 (17.2)
**Family history of celiac disease**	
Yes	66 (33.3)
**More than one child in the family with celiac disease**	
Yes	48 (24.2)
**Child’s Gender**	
Male	72 (36.4)
Female	126 (63.6)
**Time since child’s celiac diagnosis**	
<1 year	28(14.14)
1–3 years	65 (32.82)
>3 years	105 (53.03)
**Chronic diseases in addition to celiac disease**	
Yes	88 (44.4)
**Caregiver’s characteristics**	**Mean ± SD**
**Age (years)**	39.49 ± 9.84
**Average monthly income (JOD)**	541.22 ± 383.37
**Family size**	6.01 ± 1.59
**Child characteristics**	Mean ± SD
**Age (years)**	11.09 ± 4.74
**Age of the child at celiac diagnosis (years)**	8.45 ± 5.17

**Table 2:** Independent sample *t*-tests for the sociodemographic variables with the CDPCA-QoL domains (n = 198).

Variable	Category	Emotional (Mean ± SD)	Worries (Mean ± SD)	Social (Mean ± SD)
Gender of the caregiver	Female	3.50 ± 0.73	3.95 ± 0.64	2.93 ± 0.98
	Male	3.37 ± 0.74	3.90 ± 0.74	3.11 ± 1.05
	*p*-value	0.398	0.662	0.375
Marital status	Married	3.46 ± 0.73	3.95 ± 0.64	2.93 ± 0.97
	Non-married	3.70 ± 0.73	3.87 ± 0.92	3.45 ± 1.19
	*p*-value	0.293	0.683	0.074
Occupation	Yes	3.33 ± 0.80	3.82 ± 0.70	2.91 ± 1.02
	No	3.55 ± 0.68	4.01 ± 0.63	2.98 ± 0.97
	*p*-value	0.056	0.058	0.622
Caregiver with celiac disease	No	3.46 ± 0.74	3.98 ± 0.66	2.92 ± 0.98
	Yes	3.56 ± 0.69	3.78 ± 0.60	3.16 ± 0.99
	*p*-value	0.465	0.108	0.194
Family history of celiac disease	No	3.42 ± 0.74	3.91 ± 0.69	2.90 ± 0.96
	Yes	3.60 ± 0.70	4.01 ± 0.58	3.08 ± 1.02
	*p*-value	0.101	0.330	0.207
More than one affected child with CD	No	3.39 ± 0.72	3.90 ± 0.64	2.84 ± 0.96
	Yes	3.75 ± 0.71	4.09 ± 0.67	3.35 ± 0.98
	*p*-value	0.003	0.088	0.002
Gender of the child	Male	3.55 ± 0.69	4.03 ± 0.60	3.07 ± 0.84
	Female	3.44 ± 0.75	3.90 ± 0.68	2.90 ± 1.06
	*p*-value	0.281	0.181	0.233
Presence of comorbid chronic conditions	No	3.41 ± 0.75	3.89 ± 0.67	2.86 ± 0.93
	Yes	3.56 ± 0.69	4.02 ± 0.62	3.08 ± 1.05
	*p*-value	0.165	0.168	0.116

Independent sample *t*-test. Values presented as mean ± standard deviation (SD).

**Table 3:** Spearman’s rho correlations between the sociodemographic variables and the CDPCA-QoL domains.

Variable	Measure	Emotional Score	Worries Score	Social Score
Age of the caregiver	Spearman’s ρ	−0.377	−0.281	−0.251
	*p*-value	<0.001	<0.001	<0.001
Family income	Spearman’s ρ	−0.264	−0.286	−0.134
	*p*-value	<0.001	<0.001	0.059
Family size	Spearman’s ρ	0.036	0.014	0.008
	*p*-value	0.613	0.848	0.915
Age of the child with CD	Spearman’s ρ	−0.042	−0.128	−0.029
	*p*-value	0.554	0.072	0.686
Age of the child at diagnosis	Spearman’s ρ	−0.062	−0.133	−0.093
	*p*-value	0.382	0.061	0.195

Note: ρ = Spearman’s correlation coefficient. Significant correlations at the 0.01 level (2-tailed).

**Table 4:** ANOVA of the sociodemographic variables with the CDPCA-QoL domains (N = 198).

Variable	Category	Emotional Score (Mean ± SD)	Worries Score (Mean ± SD)	Social Score (Mean ± SD)
Education level	School	3.68 ± 0.63	4.06 ± 0.65	3.01 ± 1.03
	Bachelor degree	3.47 ± 0.79	3.90 ± 0.66	3.07 ± 0.95
	Diploma	3.26 ± 0.69	3.89 ± 0.63	2.71 ± 0.95
	Higher studies	2.94 ± 0.67	3.63 ± 0.65	2.83 ± 0.91
	F-test	6.685	2.295	1.205
	*p*-value	<0.001	0.079	0.309
Relationship to the child	Father	3.14 ± 0.67	3.75 ± 0.71	2.78 ± 0.87
	Mother	3.54 ± 0.70	4.00 ± 0.63	2.99 ± 0.96
	Others	3.25 ± 1.01	3.60 ± 0.70	2.81 ± 1.42
	F-test	3.589	3.405	0.604
	*p*-value	0.029	0.035	0.548
Time since child celiac diagnosis	<1 year	3.64 ± 0.77	4.09 ± 0.55	2.86 ± 1.02
	1–3 years	3.52 ± 0.70	4.10 ± 0.54	2.99 ± 0.95
	>3 years	3.41 ± 0.73	3.81 ± 0.71	2.96 ± 1.01
	F-test	1.193	4.913	0.169
	*p*-value	0.306	0.008	0.845
Age of the caregiver	≤30 years	3.94 ± 0.52	4.21 ± 0.71	3.43 ± 1.03
	31–40 years	3.65 ± 0.73	4.12 ± 0.55	3.11 ± 1.06
	41–50 years	3.25 ± 0.69	3.74 ± 0.63	2.76 ± 0.86
	>50 years	3.15 ± 0.66	3.83 ± 0.79	2.57 ± 0.83
	F-test	9.58	6.49	4.82
	*p*-value	<0.001	<0.001	0.003

Abbreviations: SD, standard deviation. Notes: *p*-values < 0.05 are considered statistically significant.

**Table 5:** Predictors (domains) associated with the overall patients’ satisfaction level using MLR, Model: Stepwise.

Predictor	B	Std. Error	*t*-Test	*p*-Value	95%CI Lower	95%CI Upper
Emotional Score						
Constant	4.246	0.311	13.655	0.000	3.633	4.859
Age of the caregiver	−0.019	0.005	−3.821	<0.001	−0.029	−0.009
Family income	0.000	0.000	−3.146	0.002	0.000	0.000
Education level	−0.158	0.053	−2.998	0.003	−0.262	−0.054
Gender of the caregiver	0.276	0.137	2.019	0.045	0.006	0.545
Worries Score						
Constant	4.778	0.196	24.381	<0.001	4.392	5.165
Family income	0.000	0.000	−3.278	0.001	0.000	0.000
Age of the caregiver	−0.014	0.005	−3.031	0.003	−0.023	−0.005
Caregiver with celiac disease	−0.300	0.120	−2.508	0.013	−0.536	−0.064
Social score						
Constant	3.921	0.293	13.365	<0.001	3.343	4.500
Age of the caregiver	−0.026	0.007	−3.817	<0.001	−0.040	−0.013
More than one child with CD	0.394	0.158	2.493	0.013	0.082	0.707

## Data Availability

The original contributions presented in this study are included in the article. Further inquiries can be directed to the corresponding authors.

## Abbreviations

The following abbreviations are used in this manuscript:

CD	celiac disease
GFD	gluten-free diet
CDPCA-QoL	Celiac Disease Parent/Caregiver Quality of Life Questionnaire
HRQoL	health-related quality of life
